# Characterization of Positional Distribution of Fatty Acids and Triacylglycerol Molecular Compositions of Marine Fish Oils Rich in Omega-3 Polyunsaturated Fatty Acids

**DOI:** 10.1155/2018/3529682

**Published:** 2018-07-10

**Authors:** Huijun Zhang, Hui Zhao, Youwei Zhang, Yingbin Shen, Hang Su, Jun Jin, Qingzhe Jin, Xingguo Wang

**Affiliations:** ^1^National Engineering Research Center for Functional Food, Collaborative Innovation Center of Food Safety and Quality Control in Jiangsu Province, State Key Laboratory of Food Science and Technology, School of Food Science and Technology, Jiangnan University, 1800 Lihu Avenue, Wuxi, Jiangsu 214122, China; ^2^Yuncheng Agricultural Vocational and Technical College, 46 Hongqi East Street, Yuncheng, Shanxi 044000, China; ^3^School of Food Science and Technology, Jiangsu Food & Pharmaceutical Science College, 4 Meicheng Road, Huai'an, Jiangsu 223003, China; ^4^Department of Food Science and Engineering, School of Science and Engineering, Jinan University, Guangzhou, Guangdong 510632, China; ^5^Shenzhen University, School of Medicine, 3688 Nanhai Ave, Shenzhen, Guangdong 518060, China

## Abstract

The regiospecific characteristics of n-3 polyunsaturated fatty acids (PUFAs) in triacylglycerol (TAG) significantly affect the physicochemical and physiological properties of marine fish oils. In this study, the TAG molecular species composition and positional distributions of fatty acids were investigated in three marine fish species rich in omega-3 PUFAs (anchovy, tuna, and salmon). The regiospecific distribution of the fatty acids was measured with the allylmagnesium bromide (AMB) degradation method. The TAG compositions were analyzed with HPLC and the TAG molecular species were identified with APCI/MS. DHA was preferentially distributed at the sn-2 position of TAG, whereas EPA was evenly distributed along the glycerol backbone. The combinations of FAs, DDO, EOP, EPS, DSS, OOS, and PPS were the predominant TAG molecular species, and OOP, DOS, and DPoPo were the characteristic TAG molecules in the anchovy, salmon, and tuna, respectively. These data can be used to distinguish other marine fish species. The TAG composition categorized by TCN and ECN showed well-structured distributions, with double or triple peaks. These findings should greatly extend the use of marine fish oils in food production and may significantly affect the future development of the fish oil industry.

## 1. Introduction

The health benefits of omega-3 polyunsaturated fatty acids (n-3 PUFAs), especially eicosapentaenoic acid (EPA) and docosahexaenoic acid (DHA), are well documented in the literature. DHA in the retina has been associated with visual outcomes and in the brain with intellectual and behavioral outcomes [1, 2]. EPA is a precursor of various signaling molecules (eicosanoids) that regulate the blood flow, immune responses, and ion transport [3, 4]. EPA and DHA also prevent cardiovascular disease and Alzheimer's disease [5]. Recently, several studies have reported that n-3 PUFAs also play a critical role in alleviating cancer and inflammatory and depressive disorders [6]. Unfortunately, EPA and DHA are rarely taken by humans in their daily meals, except by those people who live near the sea, whose diets contain large amounts of n-3 PUFAs. Although *α*-linolenic acid (ALA) is considered the precursor of n-3 PUFAs, its conversion rate to EPA and DHA is extremely low [7], which makes it much less nutritionally efficient than the direct dietary intake of EPA and DHA. Therefore, dietary supplementation with n-3 PUFAs is a necessary health benefit in humans.

**Figure 1:**
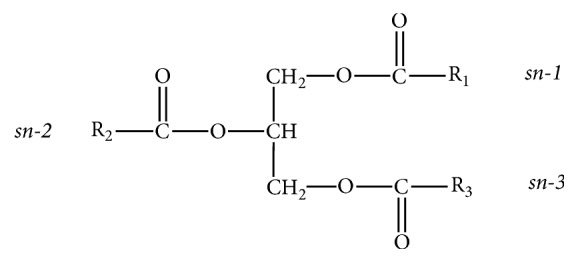
Structure of the triacylglycerol (TAG) molecule.

At present, the most common sources of n-3 PUFAs are oils of marine origin. Marine fish oils are rich in n-3 PUFAs, typically containing between 20% and 30%, and DHA and EPA account for more than 80% of these total n-3 PUFAs [8], including those derived from tuna, anchovy, and salmon. In marine fish oils, n-3 PUFAs mainly exist in the form of TAG. The TAG molecule is composed of three fatty acids (FAs) esterified to a glycerol backbone. The glycerol carbon atoms are stereospecifically numbered as sn-2 (center) and sn-1 and sn-3 (outer) (Figure 1) [9]. Different combinations of these FAs form different TAG molecular species, and the TAG molecular species composition of each oil is unique, forming complex TAG mixtures [10].

Furthermore, the characteristic effects of TAGs on digestion, absorption, and transportation are closely associated with the present TAG molecular species and their fatty-acid-binding positions [11–13]. Dietary lipids are mainly absorbed as sn-2 monoacylglycerols (2-MAGs) produced by pancreatic lipase hydrolysis of TAGs. Their absorption into enterocytes is followed by their reesterification and incorporation into chylomicrons. Therefore, the absorbed TAG molecules retain their FAs in the sn-2 positions (as in the dietary TAGs), whereas the FAs in the sn-1 and sn-3 positions are released and substituted with endogenous FAs [14, 15]. The chain lengths, degrees of unsaturation, and positional distributions of the FAs in the TAG molecule significantly affect its nutritional value and physiological functions [16, 17]. These variations reflect important biochemical and metabolic differences. Several investigations of the stability of PUFAs in regiospecific positions in TAG have shown that TAGs with PUFAs bonded to the inner sn-2 position are more stable for oxidative degradation and thermal polymerization than those esterified in the outer sn-1 and sn-3 positions [18]. The region-distribution of FAs in the sn positions of TAG molecules is the principal factor determining the physicochemical properties and phase behaviors of fats and oils [19, 20]. Therefore, to expand the use of marine fish oils in food and pharmaceutical applications, it is important to determine the compositions of the molecular species and the positional distributions of FAs in TAGs.

To the best of our knowledge, little detailed data is available on the regiospecificity of the FAs in the TAGs in marine fish oils and particularly the structures of TAGs containing PUFAs such as DHA and EPA. Therefore, the main objective of this study was to extend our knowledge of the characteristic positional distributions of TAG FAs and the TAG compositions of marine fish oils rich in n-3 PUFAs. This should improve the exploitation potentials of these valuable and underutilized resources.

## 2. Materials and Methods

### 2.1. Materials and Chemicals

Three species of marine fish oil, extracted from the anchovy, tuna, and salmon, were donated by Zhonghai Ocean Technology Co., Ltd (Jiangsu, China). Silica gel GF254 thin-layer chromatography (TLC) plates were purchased from Haiyang Chemicals Co., Ltd (Qingdao, China). The chemicals used for high-performance liquid chromatography (HPLC) were of chromatographic-grade purity and were purchased from J&K Chemical Scientific Co., Ltd (Shanghai, China). Other chemicals were of analytical grade and were purchased from Sinopharm Chemical Reagent Co., Ltd (Beijing, China). Forty fatty acid methyl ester (FAME) standards and allylmagnesium bromide (AMB) were purchased from Sigma-Aldrich (St. Louis, MO, USA).

### 2.2. Analysis of the FA Compositions of TAGs

#### 2.2.1. Methylation of TAGs

The FAMEs of the three marine fish TAGs were prepared with the method described by Birch et al. [21], with minor modification. Briefly, screw-capped tubes containing 50 *µ*g of TAG and 200 *µ*L of 10% BF_3_-methanol and 0.5 mol/L methanolic potassium hydroxide were incubated at 100°C for 30 min, after which 0.5 mL of n-hexane and 1.5 mL of deionized water were added. The mixture was centrifuged at 3,000 g for 5 min. The upper phase was collected and 1.5 mL of deionized water was added to it. The tube was vortexed and centrifuged. The processes of washing, mixing, and centrifuging were repeated three times. Finally, anhydrous sodium sulfate was added, and the tube was centrifuged at 3,000 g for 5 min before the upper layer was collected for gas chromatographic (GC) analysis.

#### 2.2.2. GC Analysis

The FAs composition was analyzed as described by Li et al. [22], with minor modification. A gas chromatograph (Agilent 7820A, USA) equipped with an autosampler, a flame ionization detector, and an ionic liquid capillary column (TRACE™ TR-FAME, 60 m × 0.25 mm × 0.25 *µ*m; Thermo Fisher, USA) was used. The oven temperature was maintained at 60°C for 90 min. The temperature of both the injector and the detector was set to 250°C. The analysis was performed with the temperature gradient program: 60°C for 3 min; 5°C/min to 175°C (held for 15 min); and then 2°C/min to a final temperature of 220°C for 10 min. Nitrogen was used as the carrier gas, at a flow rate of 1.2 mL/min, a split ratio of 1:100, detector gas hydrogen at 30 mL/min, air at 400 mL/min, and nitrogen at 25 mL/min. The GC peaks were identified by comparing their retention times with those of the corresponding standards.

### 2.3. Positional Distributions of FAs in TAGs

#### 2.3.1. Grignard Degradation

The samples were partially degraded with AMB according to the method adapted by Xu et al. [23], with slight modification. About 30 mg of each sample was dissolved in diethyl ether (10 mL) in a 50 mL round-bottomed flask. AMB (0.2 mL) was added with vigorous stirring, and the degradation reaction was stopped after 1 min with 10 mL of acid buffer (0.3 M HCl in 0.4 M boric acid). The mixture was transferred to a methylation tube, and the water phase was removed. The diethyl ether extract was washed twice with boric acid and dried with anhydrous sodium sulfate. After the sample was transferred to another small methylation tube, the ether was evaporated under nitrogen and the TAG was redissolved in 150 *µ*L of ether.

#### 2.3.2. Separation of sn-2 MAGs with TLC

Sn-2 monoglyceride (sn-2 MAG) was separated with thin-layer chromatography (TLC). TLC plates were coated with 0.4 M boric acid, air-dried overnight, and stored in a desiccator until use. The sn-2 MAG fraction was separated with TLC on boric-acid-impregnated silica gel plates with 100 mL of developing solvent, chloroform: acetone (90:10). The plates were developed twice for 45 min each, with a 10-min drying period. The sn-2 MAG band was removed and extracted three times with 1 mL of diethyl ether. The corresponding bands were identified, scraped off, and extracted three times with diethyl ether.

#### 2.3.3. Methylation of sn-2 MAG

Sn-2 MAG was methylated with potassium hydroxide with the IUPAC method [23], with slight modification. The diethyl ether in the extract was first evaporated with nitrogen. The sn-2 MAG was redissolved in 0.3 mL of heptane and methylated with 30 *µ*L of 2 M KOH in methanol solution. The supernatant was transferred to GC vials.

#### 2.3.4. GC Analysis of FAMEs

The FAMEs were analyzed with the GC analysis procedure and parameters described in the previous section.

### 2.4. Separation of TAG Molecules with Reversed-Phase-HPLC with an Evaporative Light-Scattering Detector (RP-HPLC-ELSD)

The TAG samples (50 mg) were separated on TLC plates (20 × 20 cm^2^, 0.25 mm thickness) with a mixture of n-hexane: diethyl ether: acetic acid (80:20:1, v:v:v) for molecular species analysis. The bands corresponding to the TAGs were scraped off and recovered by extraction with n-hexane. The TAG molecular species were analyzed with RP-HPLC-ELSD.

The recovered TAGs (30 mg) were dissolved in 1 mL of n-hexane, and a 10 mL portion of this solution was injected into the HPLC apparatus equipped with an ELSD (Waters, USA). The ELSD was set at 55°C, with a nitrogen nebulizer gas at a flow rate of 1.8 mL/min. Separation was performed on a 250 × 4.6 mm i.d. 5 *μ*m Lichrospher C18 column with a 4 mm × 4 mm i.d. guard column of the same material (Hanbon Science & Technology Co., Ltd, Jiangsu, China). The samples were eluted with a binary gradient of acetonitrile (A) and isopropanol (B) at a flow rate of 0.8 mL/min, with a linear gradient of solvent A from 70% to 60% in the first 30 min and then to 55% in 40 min, held at 55% for 20 min, and then increased to 70% in 5 min. The column temperature was 30°C. The sample concentration was 20 mg/mL in hexane and the injection volume was 10 *μ*L.

### 2.5. Identification of TAG Molecular Species with HPLC-Atmospheric Pressure Chemical Ionization/Mass Spectrometry (HPLC-APCI/MS)

The TAG molecular species were identified with HPLC-APCI/MS. The analyses were performed in a solvent delivery system coupled to a Micromass ZQ Mass Spectrometer (Waters) fitted with an APCI source, with full-scan acquisition. Data acquisition, processing, and instrument control were managed with the Xcalibur™ software (Thermo Scientific). The instrumental conditions were vaporizer temperature 400°C, capillary voltage 6.0 kV, and corona voltage 40 V. The spectra were obtained over the range of m/z 80–2000, with a scan time of 1.0 s.

### 2.6. Statistical Analysis

At least three (n ≥ 3) samples of each marine fish oil were used for the analysis. All analyses were conducted in triplicate, and the means ± standard deviations were calculated with the Microsoft Office statistical software. One-factor ANOVA and a* post hoc* test (Tukey–Kramer) were conducted to determine the significance of the differences among groups at p < 0.05.

## 3. Results and Discussion

### 3.1. FA Composition of TAGs

The FA compositions of the TAGs from anchovy, salmon, and tuna oils are shown in Table 1. As expected, EPA and DHA were the major PUFAs, myristic acid (C14:0), palmitic acid (C16:0), and stearic acid (C18:0) were the main saturated FAs (SFAs), and palmitoleic acid (C16:1) and oleic acid (C18:1) were the major monounsaturated FAs (MUFAs) in the marine fish TAGs. Although the FA compositions were similar, significant differences in the major FA contents were observed between the three marine fish oils.

Among the SFAs, the levels of C16:0 (35.90%) were significantly higher in anchovy than in salmon or tuna, whereas the level of C18:0 (10.51%) was highest in salmon. Among the MUFAs, the most abundant FA in salmon was C18:1 (32.92%), whereas C16:1 (11.73%) was most abundant in tuna. Among the PUFAs, the level of DHA (C22:6) was clearly higher in tuna (21.94%) than in the other two species, whereas the EPA content was highest in salmon (17.00%). EPA and DHA accounted for 20%–30% of the total FAs and for > 50% of the total unsaturated FAs. It is well known that ALA acts as the precursor of the longer-chain (LC) n-3 PUFAs and can be converted to EPA or DHA. However, marine fish species are incapable of desaturating and elongating ALA to LC-PUFAs because the activity of their delta-6 desaturase enzyme is low [24]. Higher levels of PUFAs are closely related to a greater consumption of microalgal species rich in PUFAs, which can result in high contents of both EPA and DHA [25].

### 3.2. Distribution of FAs at sn-2 Positions in TAGs

The distributions of the major PUFAs, MUFAs, and SFAs in the sn-2 positions of the fish TAGs considered here are shown in Table 2. The stereospecific positions of the FAs displayed both similarities and differences across the three marine fish species. Generally, EPA was distributed almost equally along the glycerol backbones, whereas DHA was preferentially distributed in the sn-2 positions of the TAG molecules. In this study, the marine fish oils investigated displayed characteristics more or less similar to those of deep-sea fish oils, which are currently used to manufacture DHA and EPA supplements and nutraceuticals in the food and pharmaceutical industries. Kralovec et al. reported that, in most commercial fish oils, EPA has no preferred positional distribution, whereas DHA prefers the sn-2 position in the TAG molecule [26]. Similar data have been reported for most marine fish oils. He et al. measured the positional distributions of FAs in the TAG from anchovy with pancreatic lipase (PL) hydrolysis and found that 61.18% of the EPA was attached at the sn-1+3 positions, whereas 71.31% of the DHA was bound at the sn-2 position [27]. This indicates that EPA was almost equally assigned to each position, whereas DHA was preferentially allocated to the sn-2 position. Akanbi et al. used ^13^C nuclear magnetic resonance (NMR) spectra to analyze the major FAs in anchovy oil and found EPA predominantly in sn-1 and sn-3 position, whereas the DHA residues were concentrated in sn-2 position [28]. Using ethyl magnesium bromide deacylation (EMB), Ando et al. reported that more than 50% of DHA was located at the sn-2 position. Mbatia et al. also demonstrated that, in salmon oil, 28 mol% EPA and 57 mol% DHA were present in the sn-2 position [29]. Standal et al. used high-resolution ^13^C NMR spectroscopy to show that DHA was concentrated in the sn-2 position in Atlantic salmon TAG, whereas EPA was randomly distributed in all three positions [30]. Previous studies have suggested that FAs are arranged and distributed to maximize the thermodynamic and kinetic stability of fish bodies in their native environments (based on seawater temperature and geographic location) [21, 25, 31]. The preferential distribution of DHA at the sn-2 position of TAG might be most stable when marine fish are moving in different areas of the sea at different environmental temperatures.

In general, the total SFAs and MUFAs are present at the sn-1 and sn-3 positions, but individual SFAs and MUFAs can occur at various sites. In this study, the saturated residues were assembled differently in the marine fish TAGs. For instance, the C18:0 and C16:0 residues occurred nearly equally at the terminal sn-1 and sn-3 sites and in the middle sn-2 position, respectively, with the exception of C18:0 in anchovy. The percentage of C14:0 differed in each fish species. It was mainly esterified in the sn-1 and sn-3 positions (14.16%) in tuna, but with a preference for the sn-2 position (43.25%) in anchovy, whereas in salmon, it was equally distributed across all three positions (34.63% each). In contrast, MUFAs C18:1 and C16:1 predominantly occurred at the terminal sn-1 and sn-3 positions, except for C16:1 in anchovy, which appeared to be equally distributed between sites.

### 3.3. Separation of TAG Molecular Species

Several approaches to the separation of TAGs have been developed, depending on the characteristics of the fats and oils involved. Chromatographic separation is the most basic way to resolve molecular species and provides information on the combinations of FAs in the TAG molecule. High-temperature GC (HT-GC) and HPLC are the most common chromatographic separation techniques and are frequently used to separate TAG molecules from vegetable oils and animal fats. Because the separation of TAGs by HT-GC is according to the total carbon number (TCN), TAGs with the same TCN cannot be efficiently separated with GC. Thermal instability and oxidation are the major problems that arise during the HT-GC analysis of TAGs containing PUFAs [32]. In contrast, TAGs are separated by HPLC based on the equivalent carbon number (ECN), which is calculated with the formula: ECN = TCN − (2 DB), where DB is the number of double bonds in the FAs in each TAG. Therefore, HPLC separation is based on both the FA chain length and the number of double bonds in the TAG molecule [33]. Hence, individual TAG molecules can be separated with HPLC. The retention time (Rt) of TAG increases as the chain length increases and decreases as the number of double bonds increases. TAGs with smaller ECNs elute earlier, whereas TAGs with larger ECNs elute later. In our study, the elution order followed this principle. Nonaqueous eluting solvent mixtures have been successfully used in the reversed-phase- (RP-) HPLC separation of TAGs [34]. The detector type also affects the separation outcome. Chamila et al. reported that when using evaporative light-scattering detector (ELSD), the abundant compounds in a sample are more apparent, because large and small amounts of TAGs can be distinguished according to the peak ratios of the chromatogram [25]. This characteristic is suitable for our purpose of identifying the dominant TAG molecular species. Based on the considerations discussed above, an RP-HPLC-ELSD system was used in this study. To improve the separation of the TAGs in marine fish oils, several pairs of solvents and various gradients were tested. After the chromatographic conditions were optimized, the best results were obtained with a solvent mixture of acetonitrile (A) and isopropanol (B), with a complex multistage elution gradient. This strategy improved the resolution by minimizing coelution.

The main TAGs in the marine fish oils were successfully separated and large amounts of TAGs were detected as relatively intense peaks on RP-HPLC-ELSD. Approximately 40 TAG species were eluted into different peaks with RP-HPLC and about 30 TAG molecules were identified with APCI-MS (listed in Table 3). These TAG species ranged from TCN48 to TCN66 and ECN30 to ECN54.

### 3.4. Identification of TAG Molecular Species

**Figure 2:**
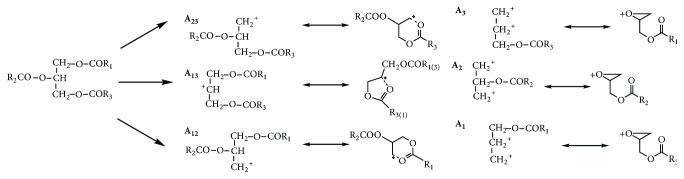
Fragment ions of TAG obtained with atmospheric pressure chemical ionization (APCI).

Mass spectrometry is a commonly used means of resolving molecular structures and can be used to determine the molecular masses of TAGs. Electrospray ion (ESI) and APCI are soft ionization techniques that generate intact TAG molecular ions and produce fragments that are useful for structural characterization when combined with MS [35, 36]. However, ESI is suitable for the analysis of polar macromolecular compounds, like proteins, peptides, etc., or relatively high-molecular-weight and poorly stable compounds [37], whereas APCI is mainly used to analyze medium-polarity or nonpolar compounds, with strong ionization efficiency [38]. Therefore, APCI-MS is usually used to identify molecular TAG species. Both positive and negative ionization modes were used to generate intact TAG molecular ions in this study, because the TAG chromatographic spectra from complex marine oils containing LC-PUFAs are usually distributed over a large range, compared with those of simple terrestrial oils. Therefore, APCI was used in both the positive and negative ionization modes to detect PUFA-rich TAGs. In the positive ionization mode, the mechanisms involved are protonation, adduct formation, and charge transfer, whereas in the negative ionization mode electron capture and anion attachment are the primary mechanisms of ion formation [25]. The FAs in the TAG molecules were identified by observing the characteristic fragment ions in the APCI mass spectra (Figure 2), together with the pseudomolecular ions [M+H]^+^ generated in the positive and negative ionization modes. The fragment ions usually observed are of three types: acyl ions [R_3_CO]^+^, MAG ions [M-R_1_COO-R_3_CO]^+^, and diacylglycerol (DAG) ions [M-R_3_COOH]^+^, where R represents an aliphatic hydrocarbon chain [39]. Although not all these ions were observed with APCI, there were enough DAG ions ([M-R_1_COOH]^+^, [M-R_2_COOH]^+^, or [M-R_3_COOH]^+^) to identify each possible pair of FAs in a particular TAG.

We successfully identified 30–40 major TAG molecular species in these three marine fish oils with HPLC–APCI-MS. Supplementary Tables 1–3 show the TAG pseudomolecular [M+H]^+^ and DAG fragmentation ions observed for the major TAG species in the marine fish oils. There were three types of combinations of FAs. When only one FA was present in the TAG molecule, such as DDD (m/z 1023.7) or EEE (m/z 945.7), only one acyl ion RCOO^−^ was observed at m/z 327.4 (D^−^) or m/z 301.5 (E^−^) in the negative- and positive-ion-mode spectra. However, when two different FAs were present in the TAG molecule, for example, in the case of DDO or EEO, two different acyl ions RCOO^−^ were observed at m/z 327.4 (D^−^) or m/z 301.5 (E^−^) and m/z 281.4 (O^−^) in the negative-ion-mode spectra because the [ODD+H]^+^ (m/z 978.1) molecules consisted of different FAs. Therefore, two [M-RCOO]^+^ ions were present, corresponding to the loss of DHA (m/z 649.7 or m/z 623.5) and oleic acid (m/z 695.4), resulting in OD^+^ or OE^+^ and DD^+^ or EE^+^ ions in the positive-ion-mode spectra, respectively. When three different FAs were present in the TAG molecule, such as [DOS+H]^+^ (m/z 933.7), the mass spectrum showed three types of [M-RCOO]^+^ ions at m/z 649.5, m/z 651.5, and m/z 605.5, corresponding to the loss of stearic acid to generate DO^+^, the loss of oleic acid to generate DS^+^, and loss of DHA to generate OS^+^, respectively. In negative ionization mode, the appropriate ion peak signals corresponded to FAs m/z 283.3 (S^−^), m/z 281.3 (O^−^), and m/z 327.4 (D^−^), respectively. Likewise, the mass spectrum of [EPS+H]^+^ (m/z 881.7) showed three types of [M-RCOO]^+^ ions, at m/z 597.50, m/z 625.53, and m/z 579.55, corresponding to the loss of stearic acid to generate EP^+^, the loss of palmitic acid to generate ES^+^, and the loss of EPA to generate PS^+^, respectively. In negative ionization mode, the appropriate ion peak signals corresponded to FAs m/z 283.26 (S^−^), m/z 255.23 (P^−^), and m/z 301.22 (E^−^), respectively. Thus, the mass spectra of the TAG molecular species showed the characteristic mass spectra of the DAGs corresponding to the loss of the appropriate FAs.

### 3.5. TAG Molecular Species Compositions

As mentioned above, the TAG species were separated with RP-HPLC and identified with APCI-MS. The major TAGs identified in the marine fish oils were expressed as percentages of the total TAGs (Table 3). Although the marine fish oils showed wide-ranging distributions of TAG species, several major TAG molecules accounted for over half of the total TAG content. Among these, TAG molecules with only a single FA were not abundant in the marine fish oils. Most TAG molecules contained two or three different FAs. In the three marine fish oils, combinations of FAs DDO, EMM, EOP, EPS, DSS, OOS, and PPS constituted the predominant TAG molecular species. The TAG species were roughly similar in these three marine fish oils, but the content of each TAG species differed significantly, as did the presence of characteristic TAG molecules.

In the anchovy, the predominant TAG molecule was EOP (11.17%), followed by DDO (9.70%) and EPoM (8.74%). These top three TAG species accounted for up to 30% of the total TAGs. It is noteworthy that OOP accounted for 3.41% of the total TAG, which was not high and differed from the OOP levels in the other two marine fish oils. In the salmon, the predominant TAG was PPS (12.18%), followed by DSS (11.67%), DOS (10.47%), and OOS (8.50%), which distinguished salmon oil from the oils of the other fish species. These three TAGs accounted for more than one-third of the total TAGs in salmon oil. These data confirm that salmon contains much more stearic and oleic acids than anchovy or tuna. Compared with the other two fish species, the tuna TAGs showed a more complex molecular species composition. The dominant TAG was EPS (17.39%), followed by EMM (12.26%), and these two TAG molecules accounted for nearly 30% of the total TAGs. However, the dominant TAG in the oil from the belly and skeletal muscles of the tuna was PDD (20.8% and 15.8%, respectively), followed by POD (9.7% and 13.0 %, respectively) [25]. More than 5% of TAGs included DPS (7.27%), DOP (6.10%), and DPoPo (6.56%). The content of DPoPo was clearly higher than in the anchovy or salmon oil because the content of palmitoleic acid (Po) was higher in the tuna oil than in the anchovy and salmon oil. However, in cod liver oil, the dominant TAG is PPoO, whereas in saury oil PED is the dominant TAG [40]. These discrepancies may be attributable to the different species, sexes, diets, seasons, and locations involved in different studies.

### 3.6. Distribution of TAG Molecular Species according to TCN and ECN

**Figure 3:**
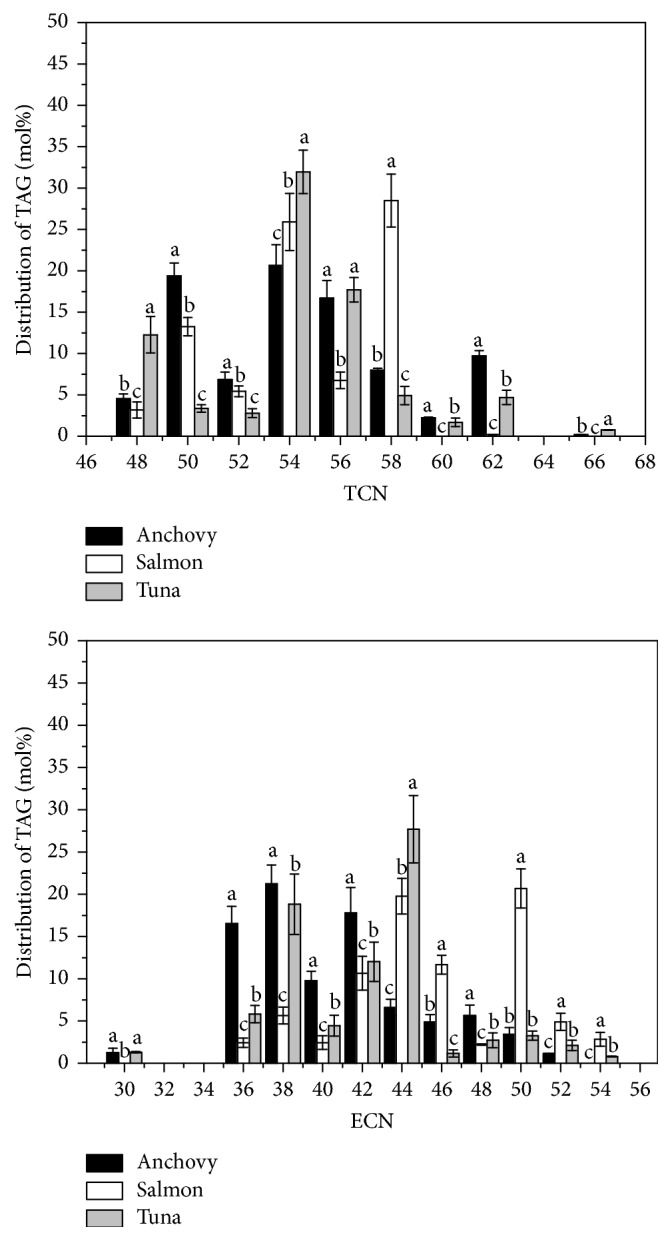
Distributions of TAGs according to total carbon number (TCN) and equivalent carbon number (ECN).

The TAG molecular compositions of the marine fish oils varied quite widely. The characteristic TAG species in the oils are summarized in histograms in Figure 3. Figures 3A and B present the TAG groups classified by TCN and ECN, respectively, and show that the TAGs are concentrated within TCN48–TCN58 and ECN36–ECN46.

In these three fish species, the TAGs showed well-structured double or triple peaks for TCN. In the anchovy (black), the TAGs were mainly distributed in TCN50, ECN54, and ECN56, and these three groups account for 56.73% of the total TAGs. These three bands principally consisted of EPoM (8.74%), EOP (11.17%), and DPoO (6.94%), which contained EPA, DHA, palmitic acid, palmitoleic acid, and oleic acid. In the salmon (white), the TAGs were enriched in TCN54 and TCN58, which accounted for 28.49% and 25.91% of the total TAGs, respectively. These two groups were chiefly represented by combinations of EPA, DHA, oleic acid, stearic acid, and palmitic acid and consisted of DSS (11.67%), DOS (10.47%), OOS (8.50%), and EOP (4.54%). In the tuna (gray), the TAGs were enriched in TCN48, TCN54, and TCN56. TCN54 was most common, accounting for almost one-third of the total TAGs (31.97%), and mainly consisted of EPS (17.39%) and DPoPo (6.56%). Briefly, TCN54 was the most frequently represented group in the TAGs of the three marine fish oils. The most frequently represented TAGs were predominantly combinations of EPA or DHA with SFAs and MUFAs containing 16 or 18 carbon atoms. Furthermore, specific TAGs were representative of each fish species and could be used to distinguish different marine fish oils, because their relative contents are very significantly higher in one species than in the others: EOP in anchovy, EPS in salmon, and OOS in tuna.

The distribution of TAGs was also characteristic of each marine fish oil. Tuna oil contained a greater proportion of TCN48 TAGs than salmon or anchovy oil, whereas salmon oil was richer in TCN58 TAGs than were the other two marine fish species. The percentage of TCN50 TAGs was higher in both anchovy and salmon oils than in tuna oil, whereas the percentage of TCN56 TAGs was significantly higher in anchovy and tuna than in salmon.

The TAGs also showed well-structured double or triple modal distributions according to ECN. In anchovy (black), the TAGs were mainly ECN36, ECN38, and ECN42, and these three groups accounted for 55.52% of the total TAGs and principally consisted of DDO (9.70%) and EPoM (8.74%). In salmon (white), the TAGs were enriched in ECN44 (19.77%) and ECN50 (20.68%) and chiefly consisted of DOS (10.47%), OOS (8.50%), and PPS (12.18%). In tuna (gray), the TAGs were mainly enriched in ECN44 (27.68%), followed by ECN38 (18.82%). These two groups accounted for nearly 50% of the total TAGs and mainly consisted of EPS (17.39%) and EMM (12.26%). When these marine fish TAGs were compared, anchovy oil had a higher content of ECN36 TAGs than salmon or tuna, whereas salmon had a higher content of ECN50 TAGs than the other two fish species. The percentage of ECN44 TAGs was markedly higher in salmon and tuna than in anchovy, whereas the percentage of ECN38 TAGs was distinctly lower in salmon than in anchovy or tuna. The three marine fish oils were abundant in ECN42 TAGs, which constituted > 10% of all three oils.

Panels A and B in Figure 3 present very similar profiles, although panel B has lower ECN values (≤ 50) and higher TCN values (≥ 50), indicating that the most abundant TAGs in these marine fish have a high degree of unsaturation. However, the salmon profiles had a peak at ECN50, suggesting that the most abundant TAGs are either saturated or monounsaturated.

## 4. Conclusion

In this study, the molecular TAG species present in anchovy, tuna, and salmon oils were first separated, identified, and discussed in detail, together with the regiospecific distributions of their FAs. Both the similarities and differences in the TAGs of these marine fish species were identified. DHA was the main esterified FA in the sn-2 position, whereas EPA was evenly incorporated at each position of the TAG molecules. Combinations of DDO, EMM, EOP, EPS, DSS, OOS, and PPS were the predominant TAG species, and OOP, DOS, and DPoPo were the characteristic TAG molecules, and these should be useful in distinguishing the three marine fish species. Management of the data according to TCN and ECN allowed the molecular TAG species to be classified and suggested that the distributions of TAGs display well-structured double or triple peaks. The TCN54 group was enriched in the TAGs and predominantly consisted of combinations of EPA or DHA with SFAs and MUFAs containing 16 or 18 carbon atoms. Therefore, the data management approach to TAG molecules based on TCN and ECN is useful in identifying the main features of different oils and allows both the differences and similarities of the samples to emerge. The lower ECN values (≤ 50) and higher TCN values (≥ 50) indicated that the most abundant TAGs had a high degree of unsaturation. Our findings should greatly extend the utilization of marine fish oils in food production and may significantly affect the future development of the fish oil industry.

## Figures and Tables

**Table 1:** Major fatty acid compositions of TAGs in marine fish oils^a^.

		**Salmon**	**Anchovy**	**Tuna**
**fatty acid**	**symbol**	%	%	%
**C14:0**	**M**	7.88±1.21	12.24±0.98*∗*	9.46±1.06
**C16:0**	**P**	14.74±1.98	35.90±2.06*∗∗*	20.00±2.05
**C16:1**	**Po**	6.96±0.68	8.33±0.56	11.73±0.99*∗*
**C18:0**	**S**	10.51±0.64*∗∗*	4.75±0.35	3.97±0.08
**C18:1**	**O**	32.92±2.01*∗∗*	16.19±1.09	22.00±1.14
**C20:5** **ω** **-3**	**E**	17.00±1.47*∗*	12.54±0.32	10.90±0.58
**C22:6** **ω** **-3**	**D**	9.99±0.25	10.04±0.68	21.94±1.39*∗∗*

^a^Values are means ± standard deviation and are expressed as mass %.

Superscript letters in a row indicate significant differences.

*∗*p < 0.05, *∗∗*p < 0.01.

**Table 2:** sn-2 positional compositions of major fatty acids in TAGs of marine fish oils^a^.

	**Salmon**		**Anchovy**		**Tuna**	
**fatty acid**	sn-2	%sn-2	sn-2	%sn-2	sn-2	%sn-2
	(mol%)	(%)	(mol%)	(%)	(mol%)	(%)
**C14:0**	6.33±0.28	31.54±0.39	12.79±0.59*∗∗*	41.87±0.27	3.12±0.25	12.86±4.88
**C16:0**	11.37±0.66	28.29±1.64	27.35±1.27	30.54±0.20	16.08±0.42	31.35±4.33
**C16:1**	4.38±1.12	24.74±1.02	6.85±0.32	32.95±0.21	5.06±0.12	16.81±0.40
**C18:0**	8.91±0.64*∗*	33.2±0.08	2.69±0.13	22.68±0.14	3.26±0.27	32.03±0.72
**C18:1**	16.15±1.02*∗*	19.27±0.74	8.71±0.40	21.58±0.14	14.68±0.43*∗*	26.01±0.54
**C20:5** **ω** **-3**	17.54±0.01	36.31±0.05	10.54±0.63	33.64±0.28	9.56±0.45	34.17±4.60
**C22:6** **ω** **-3**	12.62±1.86	50.61±3.07	11.59±0.54	49.28±0.30	25.88±0.49*∗∗*	49.00±0.86

^a^sn-2 values are means ± standard deviation and are expressed as mass %.

%sn-2 calculations were based on more than three samples with triplicate measurements per sample.

Different superscript letters in a row indicate significant differences (*∗*p < 0.05, *∗∗*p < 0.01).

**Table 3:** Triacylglyceride compositions of marine fish oils^a^.

**TAG**	**TCN**	**DB**	**ECN**	**Anchovy**	**Salmon**	**Tuna**
**EEE**	60	15	30	1.09±0.26*∗*		0.56±0.06
**DDD**	66	18	30			0.75±0.04
**EEO**	58	11	36	5.67±1.25*∗*	2.23±0.16	
**DDO**	62	13	36	9.70±1.38*∗*	0.20±0.01	4.69±0.89*∗*
**DDP**	60	12	36	1.14±0.41		1.12±0.78
**EPoPo **	52	7	38	2.64±0.56		
**DPoPo**	54	8	38		2.46±0.12	6.56±1.25*∗*
**EPoM**	50	6	38	8.74±1.11		
**EMM**	48	5	38	4.54±0.98	3.18±0.38	12.26±3.68*∗∗*
**DMM**	50	6	38	5.29±1.00		
**DPoO**	56	8	40	6.94±0.44*∗∗*	0.32±0.05	1.31±0.10*∗*
**DMO**	54	7	40	1.79±0.04*∗*	0.22±0.01	1.47±0.20*∗*
**DMP**	52	6	40	1.03±0.02	1.90±0.10	1.65±0.68
**DOO**	58	8	42		4.12±0.89	3.75±0.69
**EOP**	54	6	42	11.17±2.00*∗∗*	4.54±1.02	
**DOP**	56	7	42	4.51±0.55		6.10±1.45*∗*
**EPP**	52	5	42			1.13±0.33
**DPP**	54	6	42	2.10±0.33	1.99±0.69	1.03±0.54
**EOS**	56	6	44		2.78±0.68	3.02±0.95
**DOS**	58	7	44		10.47±2.34	
**EPS**	54	5	44	3.89±0.66	2.86±0.58	17.39±3.81*∗∗*
**DPS**	56	6	44	2.70±0.21	3.66±0.93	7.27±2.11*∗*
**ESS**	56	5	46	2.56±0.34		
**DSS**	58	6	46	2.31±0.29*∗*	11.67±2.51*∗∗*	1.16±0.02
**OOO**	54	3	48	0.54±0.01	0.53±0.04	1.70±0.12*∗*
**OOP**	52	2	48	3.17±0.58*∗∗*	0.61±0.02	
**OPP**	50	1	48	1.94±0.67	1.07±0.07	1.00±0.22
**OOS**	54	2	50		8.50±1.80*∗∗*	0.91±0.05
**PPS**	50	0	50	3.41±0.39*∗*	12.18±2.22*∗∗*	2.35±0.03
**OSS**	54	1	52	1.14±0.24	1.97±0.32	2.11±0.09
**PSS**	52	0	52		2.92±0.41	
**SSS**	54	0	54		2.84±0.54*∗*	0.80±0.11

^a^Abbreviations: ECN, equivalent carbon number; TCN, total carbon number; DB, number of double bonds.

Fatty acid symbols: M, myristic acid; P, palmitic acid; Po, palmitoleic acid; S, stearic acid; O, oleic acid; E, EPA, eicosapentaenoic acid; D, DHA, docosahexaenoic acid. Values are means ± standard deviation with triplicate measurements per sample (mass %). Different superscript letters in a row indicate significant differences (*∗*p < 0.05, *∗∗*p < 0.01).

## Data Availability

The data used to support the findings of this study are available from the corresponding author upon request.

## Supplementary Materials

Supplementary Materials Observed ions in the HPLC-APCI-mass spectra of TAGs in positive and negative ion modes. Table 1: observed ions in the HPLC-APCI-mass spectra of major salmon TAGs in positive and negative ion modes. Table 2: observed ions in the HPLC-APCI-mass spectra of major anchovy TAGs in positive and negative ion modes. Table 3: observed ions in the HPLC-APCI-mass spectra of major tuna TAGs in positive and negative ion modes.
