# Supplementary material for: Characterization of Positional Distribution of Fatty Acids and Triacylglycerol Molecular Compositions of Marine Fish Oils Rich in Omega-3 Polyunsaturated Fatty Acids
Source: Biomed Res Int. 2018 Jul 10;2018:3529682. doi: 10.1155/2018/3529682 (PMC6077587; doi:10.1155/2018/3529682)
Supplement: Supplementary Materials — Observed ions in the HPLC-APCI-mass spectra of TAGs in positive and negative ion modes. Table 1: observed ions in the HPLC-APCI-mass spectra of major salmon TAGs in positive and negative ion modes. Table 2: observed ions in the HPLC-APCI-mass spectra of major anchovy TAGs in positive and negative ion modes. Table 3: observed ions in the HPLC-APCI-mass spectra of major tuna TAGs in positive and negative ion modes. [file 3529682.f1.pdf]

Supplementary Tables——Observed ions in the HPLC－APCI-mass spectra of TAGs in positive and negative ion modes

Table 1. Observed ions in the HPLC–APCI-mass spectra of major salmon TAGs in positive and negative ion modes

TAG	TCN	UBN	ECN	Ion observed in the mass spectra of TAG												
				Molecular ion	Fragmentation ions:[(M＋H)RCOO]+						Fragmentation ions:[RCOO]$^-$					
				[M＋H]$^+$	F1	m/z	F2	m/z	F3	m/z	R1	m/z	R2	m/z	R3	m/z
**EEO**	58	11	36	925.73	**EE**	643.48	**EO**	623.51	—	—	**E**	301.22	**O**	281.25	—	—
**DDO**	62	13	36	977.76	**DD**	695.51	**DO**	649.53	—	—	**D**	327.23	**O**	281.25	—	—
**DPoPo**	54	8	38	875.71	**DPo**	621.50	**PoPo**	547.48	—	—	**D**	327.23	**Po**	253.2173	—	—
**EMM**	48	5	38	797.67	**EM**	569.47	**MM**	495.45	—	—	**E**	301.22	**M**	227.20	—	—
**DPoO**	56	8	40	903.74	**DPo**	621.50	**PoO**	595.48	**DO**	649.53	**D**	327.23	**Po**	253.2173	**O**	281.25
**DMO**	54	7	40	877.73	**DM**	595.48	**MO**	549.50	**DO**	649.53	**D**	327.23	**M**	227.20	**O**	281.25
**DMP**	52	6	40	851.71	**DM**	595.48	**MP**	523.48	**DP**	623.51	**D**	327.23	**M**	227.20	**P**	255.23
**DOO**	58	8	42	931.77	**DO**	649.53	**OO**	603.55	—	—	**D**	327.23	**O**	281.25	—	—
**EOP**	54	6	42	879.74	**EO**	623.51	**OP**	577.53	**EP**	597.50	**E**	301.22	**O**	281.25	**P**	255.23
**DPP**	54	6	42	879.74	**DP**	623.51	**PP**	551.51	—	—	**D**	327.23	**P**	255.23	—	—
**EOS**	56	6	44	907.77	**EO**	623.51	**OS**	605.56	**ES**	625.53	**E**	301.22	**O**	281.25	**S**	283.26
**DOS**	58	7	44	933.79	**DO**	649.53	**OS**	605.56	**DS**	651.55	**D**	327.23	**O**	281.25	**S**	283.26
**EPS**	54	5	44	881.76	**EP**	597.50	**PS**	579.55	**ES**	625.53	**E**	301.22	**P**	255.23	**S**	283.26
**DPS**	56	6	44	907.77	**DP**	623.51	**PS**	579.55	**DS**	651.55	**D**	327.23	**P**	255.23	**S**	283.26
**ESS**	56	5	46	909.79	**ES**	625.53	**SS**	607.58	—	—	**E**	301.22	**S**	283.26	—	—
**DSS**	58	6	46	935.81	**DS**	651.55	**SS**	607.58	—	—	**D**	327.23	**S**	283.26	—	—
**OOO**	54	3	48	885.79	**OO**	603.55	—	—	—	—	**O**	281.25	—	—	—	—
**OOP**	52	2	48	859.77	**OO**	603.55	**OP**	577.53	—	—	**O**	281.25	**P**	255.23	—	—
**OPP**	50	1	48	833.76	**OP**	577.53	**PP**	551.51	—	—	**O**	281.25	**P**	255.23	—	—
**OOS**	54	2	50	887.81	**OO**	603.55	**OS**	605.56	—	—	**O**	281.25	**S**	283.26	—	—
**PPS**	50	0	50	835.77	**PP**	551.51	**PS**	579.55	—	—	**P**	255.23	**S**	283.26	—	—
**OSS**	54	1	52	889.82	**OS**	605.56	**SS**	607.58	—	—	**O**	281.25	**S**	283.26	—	—
**PSS**	52	0	52	863.81	**PS**	579.55	**SS**	607.58	—	—	**P**	255.23	**S**	283.26	—	—
**SSS**	54	0	54	891.84	**SS**	607.58	—	—	—	—	**S**	283.26	—	—	—	—

TCN: total carbon number; DB: number of double bonds; ECN: equivalent carbon number; ECN = TCN − (2 × DB);

F-1, F-2, and F-3: fragmentation ions of diacylglycerols; R^1, R^2, and R^3: fragmentation ions of fatty acids.

Table 2. Observed ions in the HPLC–APCI-mass spectra of major anchovy TAGs in positive and negative ion modes

TAG	TCN	DB	ECN	Ion observed in the mass spectra of TAG												
				Molecular	Fragmentation ions:[(M＋H)RCOO]+						Fragmentation ions:[RCOO]-					
				$[M+H]^+$	F1	m/z	F2	m/z	F3	m/z	R1	m/z	R2	m/z	R3	m/z
**EEE**	60	15	30	945.70	**EE**	643.48	—	—	—	—	**E**	301.22	—	—	—	—
**DDD**	66	18	30	1023.74	**DD**	695.51	—	—	—	—	**D**	327.23	—	—	—	—
**EEO**	58	11	36	925.73	**EE**	643.48	**EO**	623.51	—	—	**E**	301.22	**O**	281.25	—	—
**DDO**	62	13	36	977.76	**DD**	695.51	**DO**	649.53	—	—	**D**	327.23	**O**	281.25	—	—
**DDP**	60	12	36	951.74	**DD**	695.51	**DP**	623.51	—	—	**D**	327.23	**P**	255.23	—	—
**EPoPo**	52	7	38	849.70	**EPo**	595.48	**PoPo**	547.48	—	—	**E**	301.22	**Po**	253.22	—	—
**EPoM**	50	6	38	823.68	**EPo**	595.48	**PoM**	521.47	**EM**	569.47	**E**	301.22	**Po**	253.22	**M**	227.20
**EMM**	48	5	38	797.67	**EM**	569.47	**MM**	495.45	—	—	**E**	301.22	**M**	227.20	—	—
**DMM**	50	6	38	823.68	**DM**	595.48	**MM**	495.45	—	—	**D**	327.23	**M**	227.20	—	—
**DPoO**	56	8	40	903.74	**DPo**	621.50	**PoO**	595.48	**DO**	649.53	**D**	327.23	**Po**	253.22	**O**	281.25
**DMO**	54	7	40	877.73	**DM**	595.48	**MO**	549.50	**DO**	649.53	**D**	327.23	**M**	227.20	**O**	281.25
**DMP**	52	6	40	851.71	**DM**	595.48	**MP**	523.48	**DP**	623.51	**D**	327.23	**M**	227.20	**P**	255.23
**EOP**	54	6	42	879.74	**EO**	623.51	**OP**	577.53	**EP**	597.50	**E**	301.22	**O**	281.25	**P**	255.23
**DOP**	56	7	42	905.76	**DO**	649.53	**OP**	577.53	**DP**	623.51	**D**	327.23	**O**	281.25	**P**	255.23
**DPP**	54	6	42	879.74	**DP**	623.51	**PP**	551.51	—	—	**D**	327.23	**P**	255.23	—	—
**EPP**	52	5	42	853.73	**EP**	597.50	**PP**	551.51	—	—	**E**	301.22	**P**	255.23	—	—
**EOS**	56	6	44	907.77	**EO**	623.51	**OS**	605.56	**ES**	625.53	**E**	301.22	**O**	281.25	**S**	283.26
**EPS**	54	5	44	881.76	**EP**	597.50	**PS**	579.55	**ES**	625.53	**E**	301.22	**P**	255.23	**S**	283.26
**DPS**	56	6	44	907.77	**DP**	623.51	**PS**	579.55	**DS**	651.55	**D**	327.23	**P**	255.23	**S**	283.26
**ESS**	56	5	46	909.79	**ES**	625.53	**SS**	607.58	—	—	**E**	301.22	**S**	283.26	—	—
**DSS**	58	6	46	935.81	**DS**	651.55	**SS**	607.58	—	—	**D**	327.23	**S**	283.26	—	—
**OOO**	54	3	48	885.79	**OO**	603.55	—	—	—	—	**O**	281.25	—	—	—	—
**OOP**	52	2	48	859.77	**OO**	603.55	**OP**	577.53	—	—	**O**	281.25	**P**	255.23	—	—
**OPP**	50	1	48	833.76	**OP**	577.53	**PP**	551.51	—	—	**O**	281.25	**P**	255.23	—	—
**PPS**	50	0	50	835.77	**PP**	551.51	**PS**	579.55	—	—	**P**	255.23	**S**	283.26	—	—
**OSS**	54	1	52	889.82	**OS**	605.56	**SS**	607.58	—	—	**O**	281.25	**S**	283.26	—	—

TCN-total carbon number; DB-double bond number; ECN-equivalent carbon number, ECN = TCN - (2×DB);

F-1, F-2, and F-3: fragmentation ions of diacylglycerols; R^1, R^2, and R^3: fragmentation ions of fatty acids.

Table 3. Observed ions in the HPLC–APCI-mass spectra of major tuna TAGs in positive and negative ion modes

TAG	TCN	DB	ECN	Ion observed in the mass spectra of TAG												
				Molecular ion	Fragmentation ions:[(M＋H)RCOO]+						Fragmentation ions:[RCOO]$^-$					
				$[M+H]^+$	**F1**	m/z	**F2**	m/z	**F3**	m/z	**R1**	m/z	**R2**	m/z	**R3**	m/z
**EEE**	60	15	30	945.70	**EE**	643.48	—	—	—	—	**E**	301.22	—	—	—	—
**DDD**	66	18	30	1023.74	**DD**	695.51	—	—	—	—	**D**	327.23	—	—	—	—
**DDO**	62	13	36	977.76	**DD**	695.51	**DO**	649.53	—	—	**D**	327.23	**O**	281.25	—	—
**DDP**	60	12	36	951.74	**DD**	695.51	**DP**	623.51	—	—	**D**	327.23	**P**	255.23	—	—
**DPoPo**	54	8	38	875.71	**DPo**	621.50	**PoPo**	547.48	—	—	**D**	327.23	**Po**	253.2173	—	—
**EMM**	48	5	38	797.67	**EM**	569.47	**MM**	495.45	—	—	**E**	301.22	**M**	227.20	—	—
**DPoO**	56	8	40	903.74	**DPo**	621.50	**PoO**	595.48	**DO**	649.53	**D**	327.23	**Po**	253.2173	**O**	281.25
**DMO**	54	7	40	877.73	**DM**	595.48	**MO**	549.50	**DO**	649.53	**D**	327.23	**M**	227.20	**O**	281.25
**DMP**	52	6	40	851.71	**DM**	595.48	**MP**	523.48	**DP**	623.51	**D**	327.23	**M**	227.20	**P**	255.23
**DOO**	58	8	42	931.77	**DO**	649.53	**OO**	603.55	—	—	**D**	327.23	**O**	281.25	—	—
**DOP**	56	7	42	905.76	**DO**	649.53	**OP**	577.53	**DP**	623.51	**D**	327.23	**O**	281.25	**P**	255.23
**EPP**	52	5	42	853.73	**EP**	597.50	**PP**	551.51	—	—	**E**	301.22	**P**	255.23	—	—
**EOS**	56	6	44	907.77	**EO**	623.51	**OS**	605.56	**ES**	625.53	**E**	301.22	**O**	281.25	**S**	283.26
**DOS**	58	7	44	933.79	**DO**	649.53	**OS**	605.56	**DS**	651.55	**D**	327.23	**O**	281.25	**S**	283.26
**EPS**	54	5	44	881.76	**EP**	597.50	**PS**	579.55	**ES**	625.53	**E**	301.22	**P**	255.23	**S**	283.26
**DPS**	56	6	44	907.77	**DP**	623.51	**PS**	579.55	**DS**	651.55	**D**	327.23	**P**	255.23	**S**	283.26
**ESS**	56	5	46	909.79	**ES**	625.53	**SS**	607.58	—	—	**E**	301.22	**S**	283.26	—	—
**DSS**	58	6	46	935.81	**DS**	651.55	**SS**	607.58	—	—	**D**	327.23	**S**	283.26	—	—
**OOO**	54	3	48	885.79	**OO**	603.55	—	—	—	—	**O**	281.25	—	—	—	—
**OOP**	52	2	48	859.77	**OO**	603.55	**OP**	577.53	—	—	**O**	281.25	**P**	255.23	—	—
**OPP**	50	1	48	833.76	**OP**	577.53	**PP**	551.51	—	—	**O**	281.25	**P**	255.23	—	—
**OOS**	54	2	50	887.81	**OO**	603.55	**OS**	605.56	—	—	**O**	281.25	**S**	283.26	—	—
**PPS**	50	0	50	835.77	**PP**	551.51	**PS**	579.55	—	—	**P**	255.23	**S**	283.26	—	—
**OSS**	54	1	52	889.82	**OS**	605.56	**SS**	607.58	—	—	**O**	281.25	**S**	283.26	—	—
**SSS**	54	0	54	891.84	**SS**	607.58	—	—	—	—	**S**	283.26	—	—	—	—

TCN: total carbon number; DB: number of double bonds; ECN: equivalent carbon number; ECN = TCN − (2 × DB);

F-1, F-2, and F-3: fragmentation ions of diacylglycerols; R^1, R^2, and R^3: fragmentation ions of fatty acids.
